# Comment on: “Gastric adenocarcinoma and periodontal disease: a systematic review and meta-analysis”

**DOI:** 10.1016/j.clinsp.2025.100596

**Published:** 2025-03-08

**Authors:** Shubham Kumar, Nosaibah Razaqi, Rachana Mehta, Ranjana Sah

**Affiliations:** a Center for Global Health Research, Saveetha Medical College and Hospital, Saveetha Institute of Medical and Technical Sciences, Saveetha University, Chennai, India; b Afghanistan Center for Epidemiological Studies, Herat, Afghanistan; c Faculty of Medicine, Ghalib University, Herat, Afghanistan; d Scientific Research Committee, Afghanistan Medical Students Association, Herat, Afghanistan; e Clinical Microbiology, RDC, Manav Rachna International Institute of Research and Studies, Faridabad, Haryana, India; f Department of Paediatrics, Dr. D. Y. Patil Medical College Hospital and Research Centre, Dr. D. Y. Patil Vidyapeeth (Deemed-to-be-University), Pimpri, Maharashtra, India; g Department of Public Health Dentistry, Dr. D. Y. Patil Medical College Hospital and Research Centre, Dr. D. Y. Patil Vidyapeeth (Deemed-to-be-University), Pimpri, Maharashtra, India; h Medical Laboratories Techniques Department, AL-Mustaqbal University, Babil, Iraq

We read with great interest the recently published article titled “Gastric Adenocarcinoma and Periodontal Disease: A Systematic Review and Meta-Analysis”, which offers significant insights into the potential linkage between periodontal disease and gastric adenocarcinoma [1]. The authors' rigorous approach to synthesizing the existing literature is commendable and contributes substantially to our understanding of this complex relationship. However, we wish to suggest several methodological enhancements that could strengthen the findings and implications of this systematic review.

The methodological rigor of the study would have been enhanced by a more detailed presentation of the quality assessment outcomes. While the Newcastle-Ottawa Scale (NOS) was appropriately used for quality assessment, reporting the specific scores for each included study would provide clarity regarding their individual contributions to the meta-analysis. This transparency would allow readers to better assess the weight and relevance of each study's findings within the overall analysis. Moreover, considering the observed heterogeneity among the included studies, meta-regression would have been a suitable method to explore potential sources of this variability [2]. Such an analysis could help clarify under what conditions periodontal disease most strongly predicts gastric adenocarcinoma risk, considering variables such as study design, sample size, or participant demographics.

In terms of publication bias, the authors could have enhanced their analysis by employing the trim and fill method alongside the funnel plot and Egger's test already utilized. This approach not only assesses the presence of bias but also adjusts for it, potentially providing a more accurate estimate of the effect size. Furthermore, the use of prediction intervals along with confidence intervals in the presentation of the meta-analysis results would give readers a better understanding of the expected range of true effects in similar future studies, enhancing the interpretation of the data given the clinical diversity and methodological variability among the included studies [3,4].

Implementing the GRADE (Grading of Recommendations Assessment, Development, and Evaluation) approach alongside the NOS for assessing the quality of evidence would provide a structured and transparent method for rating the quality of evidence and the strength of recommendations. This approach is particularly crucial in systematic reviews where the clinical applicability of findings must be communicated clearly to practitioners and policymakers [5].

While the article provides valuable insights into the relationship between periodontal disease and gastric adenocarcinoma, addressing these methodological considerations could significantly enhance the validity and applicability of the findings. Such enhancements would ensure that the conclusions drawn are based on a robust and comprehensive synthesis of available evidence, thereby better informing clinical practice and future research directions.

## Ethical approval

Not Applicable.

## Authors’ contributions

SK, RM, RS, and NR critically and provided comments on methodological aspects, SK, NR, and RS have written the edited the draft.

## Financial support

No funding was received for this research.

## Declaration of competing interest

The authors declare no conflicts of interest.
